# 血友病合并抑制物患者应用凝血酶原复合物序贯重组人凝血因子Ⅶa围术期治疗1例

**DOI:** 10.3760/cma.j.cn121090-20250104-00006

**Published:** 2024-12

**Authors:** 雪雯 宋, 梦娟 李, 冰洁 丁, 烽迪 王, 俊霞 胡, 静 张, 虎 周

**Affiliations:** 郑州大学附属肿瘤医院（河南省肿瘤医院），郑州 450008 The Affiliated Cancer Hospital of Zhengzhou University & Henan Cancer Hospital, Zhengzhou 450008, China

患者，男，42岁，体重53 kg。患者40年前无明显诱因出现关节出血，不易止血，至医院就诊，显示凝血功能异常，凝血因子Ⅷ<1％，明确诊断为“血友病A型（重型）”，予血浆、冷沉淀及人凝血因子Ⅷ制品（血源、重组）按需治疗，年出血次数约50次。患者9个月前自觉凝血因子制品效果不佳，就诊于当地医院，查FⅧ抑制物滴度1.6 BU/ml，接受凝血酶原复合物（PCC）间断治疗。患者双膝、双踝、双肘关节出血，左膝关节为靶关节，为行左膝关节置换术入院。既往无特殊病史，无家族史。郑州大学附属肿瘤医院复查FⅧ抑制物滴度51.2 BU/ml，依据《世界血友病联盟血友病管理指南（第3版）》中建议的抑制物旁路途经，结合本中心经验，予重组活化凝血因子Ⅶ（rFⅦa）联合凝血酶原复合物（PCC）的序贯联合旁路治疗（SCBT）方案，第1～3天PCC 50 IU/kg每6 h 1次+重组人凝血因子Ⅶa 90 µg/kg每6 h 1次间隔应用，第4～6天PCC 30 IU/kg每8 h 1次+重组人凝血因子Ⅶa 90 µg/kg每8 h 1次间隔使用，第7～14天PCC 30 IU/kg每8 h 1次至每12 h 1次逐渐减量。顺利完成人工膝关节铰链式置换术+关节滑膜切除术，术中出血200 ml，未输注血制品，术中止血效果评估极好，术后恢复良好（图1）。术后监测D-二聚体升高，最高达3.65 mg/L，无血栓事件。术后14 d于活动后突发左膝关节出血，伴伤口渗血，HGB从术后11 d的114 g/L降至83 g/L。将PCC 1 000 IU每12 h 1次调整为PCC 1 600 IU每12 h 1次，效果不佳，并急查FⅦa活性54％，抑制物未检出，FⅩ活性41％，抑制物未检出，于术后15 d调整为PCC 2 600 IU每8 h 1次+重组人凝血因子Ⅶa 5 mg每12 h 1次×1 d，并输注血浆300 ml，术后16 d停止出血，无关节疼痛及关节渗血，术后21 d复查HGB升至116 g/L，患者于术后25 d顺利出院。

**图1:**
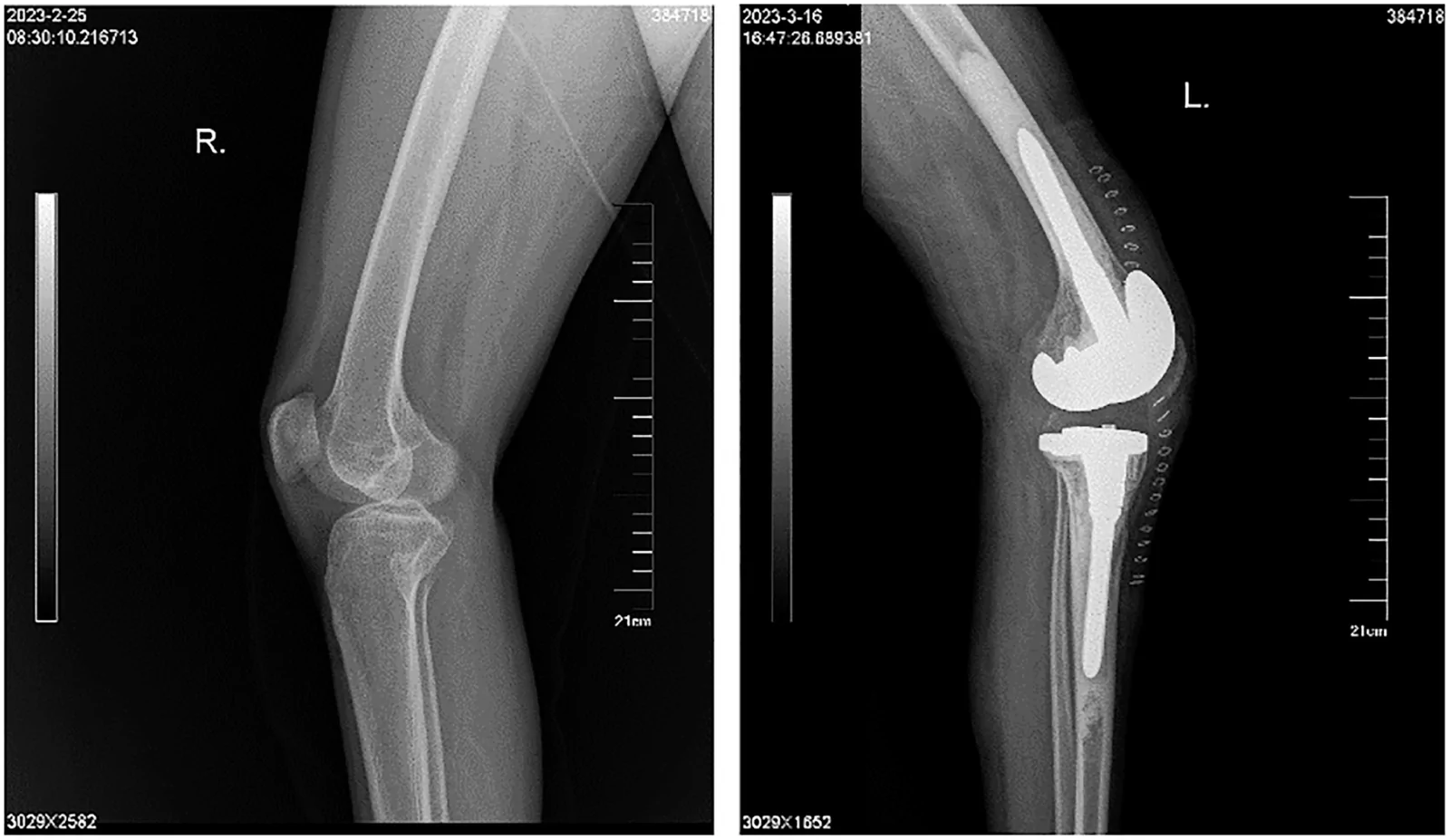
本例患者左膝关节手术前（左）后（右）检查对比

随访该患者，术后1个月顺利拆线，术后2个月可自行行走，短时快走，术后2年步态基本恢复正常，截至目前，患者在接受免疫耐受诱导治疗。

讨论：血友病是一类由凝血因子Ⅷ或Ⅸ缺乏引起的自发性出血性疾病，凝血因子类产品按需或预防性治疗为目前的主要治疗手段。然而，随着治疗的不断进步，患者生存期延长，越来越多的患者有各种手术需求，如关节手术、肿物切除等。血友病凝血因子治疗的最大难点为抑制物的形成，而血友病伴抑制物患者的手术更是一大难点，术中高出血风险、术后突破性出血、围术期血栓监测、术后感染的预防等都是需要持续关注的问题。旁路途径一直是血友病伴抑制物患者止血治疗的关键方法，而围术期凝血因子的调整目前尚无统一方法，包括凝血因子的使用剂量、减药方式、间隔时间等，均需要进一步探索。

本例患者有明确抑制物阳性病史，Bethesda法测出高滴度抑制物，应用凝血酶原复合物与重组人凝血因子Ⅶa序贯治疗的方式，成功完成手术，患者活动后出现突破性出血时，通过适当增加PCC和FⅦa制剂的方法，及时止血，且未发现血栓形成，证明该方案安全有效。

